# Preclinical Pharmacokinetics and Biodistribution of Anticancer Dinuclear Palladium(II)-Spermine Complex (Pd_2_Spm) in Mice

**DOI:** 10.3390/ph14020173

**Published:** 2021-02-23

**Authors:** Martin Vojtek, Salomé Gonçalves-Monteiro, Edgar Pinto, Sára Kalivodová, Agostinho Almeida, Maria P. M. Marques, Ana L. M. Batista de Carvalho, Clara B. Martins, Helder Mota-Filipe, Isabel M. P. L. V. O. Ferreira, Carmen Diniz

**Affiliations:** 1 LAQV/REQUIMTE, Laboratory of Pharmacology, Department of Drug Sciences, Faculty of Pharmacy, University of Porto, 4050-313 Porto, Portugal; salomemonteiro8180@gmail.com (S.G.-M.); KalivodovaSara@seznam.cz (S.K.); 2 LAQV/REQUIMTE, Laboratory of Bromatology and Hydrology, Department of Chemical Sciences, Faculty of Pharmacy, University of Porto, 4050-313 Porto, Portugal; ecp@ess.ipp.pt (E.P.); isabel.ferreira@ff.up.pt (I.M.P.L.V.O.F.); 3 Department of Environmental Health, School of Health, P.Porto, CISA/Research Center in Environment and Health, 4200-072 Porto, Portugal; 4 LAQV/REQUIMTE, Laboratory of Applied Chemistry, Department of Chemical Sciences, Faculty of Pharmacy, University of Porto, 4050-313 Porto, Portugal; aalmeida@ff.up.pt; 5 “Molecular Physical-Chemistry” R&D Unit, Department of Chemistry, University of Coimbra, 3004-535 Coimbra, Portugal; pmc@ci.uc.pt (M.P.M.M.); almbc@uc.pt (A.L.M.B.d.C.); martinscsb@gmail.com (C.B.M.); 6 Department of Life Sciences, University of Coimbra, 3000-456 Coimbra, Portugal; 7 iMed.ULisboa, Faculty of Pharmacy, University of Lisbon, 1649-003 Lisbon, Portugal; hfilipe@campus.ul.pt

**Keywords:** Pd(II)-based drugs, cisplatin, ICP-MS, metal complexes, polyamines, cancer, tissue, in vivo

## Abstract

Palladium-based compounds are regarded as potential analogs to platinum anticancer drugs with improved properties. The present study assessed the pharmacokinetics and biodistribution of a dinuclear palladium(II)-spermine chelate (Pd_2_Spm), which has previously been shown to possess promising in vitro activity against several therapy-resistant cancers. Using inductively coupled plasma-mass spectrometry, the kinetic profiles of palladium/platinum in serum, serum ultrafiltrate and tissues (kidney, liver, brain, heart, lungs, ovaries, adipose tissue and mammary glands) were studied in healthy female Balb/c mice after a single intraperitoneal bolus injection of Pd_2_Spm (3 mg/kg bw) or cisplatin (3.5 mg/kg bw) between 0.5 and 48 h post-injection. Palladium in serum exhibited biphasic kinetics with a terminal half-life of 20.7 h, while the free palladium in serum ultrafiltrate showed a higher terminal half-life than platinum (35.5 versus 31.5 h). Palladium was distributed throughout most of the tissues except for the brain, with the highest values in the kidney, followed by the liver, lungs, ovaries, adipose tissue and mammary glands. The in vitro cellular accumulation was also evaluated in breast cancer cells, evidencing a passive diffusion as a mechanism of Pd_2_Spm’s cellular entry. This study reports, for the first time, the favorable pharmacokinetics and biodistribution of Pd_2_Spm, which may become a promising pharmacological agent for cancer treatment.

## 1. Introduction

**Figure 1:**
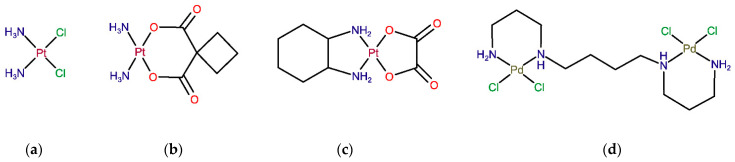
Structure of Pt(II)- and Pd(II)-based agents: Worldwide approved Pt drugs—(**a**) cisplatin, (**b**) carboplatin and (**c**) oxaliplatin. (**d**) Novel palladium(II)-based complex Pd_2_Spm.

The discovery of the anticancer properties of the platinum(II)-based drug cisplatin (*cis*-Pt(NH_3_)_2_Cl_2_) (Figure 1a) has revolutionized the therapy of solid neoplasms, namely, breast, testicular, ovarian, cervical, prostate, head, neck, bladder and lung cancers, as well as refractory non-Hodgkin’s lymphoma [1]. However, its therapeutic use is limited by inherent or acquired tumor cell resistance and dose-dependent toxicity (namely, nephrotoxicity, hepatotoxicity, neurotoxicity and myelosuppression). To overcome these limitations, several modifications of cisplatin’s structure have led to the development of carboplatin and oxaliplatin, which have also received worldwide approval for clinical use. Carboplatin (Figure 1b) is a 2nd generation platinum drug with an improved pharmacokinetic profile and tolerability, owing to the substitution of the two chloride ligands by a cyclobutanedicarboxylate group that has a slower hydrolysis rate to yield reactive species prone to bind to DNA [2]. Carboplatin is indicated in a monotherapy regimen of ovarian and small cell lung carcinomas, but it shares a cross-resistance with cisplatin in previously cisplatin-treated patients, which limits its clinical use [3]. Oxaliplatin (Figure 1c) is a 3rd generation platinum analog with a diaminocyclohexane ligand that significantly reduces its toxicity and glutathione-mediated acquired tumor cell resistance [4]. Although oxaliplatin is less reactive towards DNA than cisplatin, it was demonstrated in vitro that its crosslinks appear to be more damaging [5]. Currently, oxaliplatin in combination with 5-fluorouracil is approved for the treatment of advanced colorectal cancer.

The pharmacodynamic effects of cisplatin and other platinum agents are generally accepted to be mediated by the binding to DNA in the nucleus and mitochondria, while other non-DNA targets, such as RNA, tubulin, thioredoxin reductase, membrane-bound Na^+^/H^+^ exchanger protein [6], or even intracellular water [7], have also been reported. Upon cellular entry, the drug’s hydrolyzable ligands are substituted with water molecules, yielding highly reactive aqua species that bind to DNA (mainly through the purine´s nitrogen atoms) [8]. The formation of these drug-DNA adducts prompts DNA damage, which interferes with the replication and transcription processes and leads to cell growth impairment and cellular death [2].

Despite the indisputable role of mononuclear platinum drugs (compounds with one metal center, such as cisplatin, carboplatin and oxaliplatin) in the therapy of solid neoplasms, there is considerable interest in the development of more efficient metal-based antitumor agents that could surpass the drawbacks of conventional platinum drugs while presenting a broader therapeutic spectrum coupled to low toxicity [9]. Even though from the thermodynamic point of view, a Pt-ligand bond in conventional platinum(II) coordination compounds presents somewhat lower thermodynamic durability (≤100 kJ/mol) than single and double covalent bonds between C–C, C–N or C–O (250–500 kJ/mol), the ligand substitution is rather slow, yielding reasonably stable molecules with desired in vivo pharmacokinetic and pharmacodynamic properties [10,11]. In addition to numerous platinum compounds developed in recent decades [9], complexes containing other transition metals have also been investigated, including palladium(II) [12], gold(I), gold(II), tin(IV) [13], ruthenium(II), ruthenium(III) [14] and copper(II)-based compounds [15]. Palladium(II) complexes have received particular interest as potential Pt(II) analogs thanks to the similarities in structure and coordination chemistry between these two metal ions [12,16]. As observed for Pt coordination compounds, a successful substitution of the central atom for Pd(II) should be closely related to the nature of its ligands (including leaving groups) in order to provide suitable thermodynamic and kinetic features: thermodynamic stability coupled to low kinetic lability (to avoid instantaneous ligand exchange). In particular, biogenic polyamines, such as spermidine (H_2_N(CH_2_)_4_NH(CH_2_)_3_NH_2_, Spd) and spermine (H_2_N(CH_2_)_3_NH(CH_2_)_4_NH(CH_2_)_3_NH_2_, Spm), have been identified as suitable chelating ligands for both Pt and Pd-metal ions, allowing the formation of stable chelates with appropriate kinetic properties [17]. For instance, the dinuclear Pd(II) complex with spermine as a bridging ligand (Pd_2_Spm, Figure 1d) has shown promising in vitro anticancer activity against osteosarcoma [18], breast [19,20] and ovarian cancers [21]. Pd_2_Spm´s antineoplastic activity is attributed to unconventional DNA interactions at more than one site in the double helix via the formation of long-range interstrand adducts that trigger more severe and less repairable cell damage than the clinically used mononuclear Pt(II) compounds (e.g., cisplatin) [17,22,23]. Additionally, Pd-based compounds are expected to display higher tolerability and safety profiles than conventional Pt-based drugs [24,25], which warrants their further in vivo investigation.

Despite the promising studies that have already reported on the anticancer properties of Pd(II)-agents, information on their pharmacokinetic profile, including in vivo biodistribution, is scarce. To the best of the authors´ knowledge, the preclinical assays of padeliporfin (approved in 2017 for the vascular-targeted photodynamic therapy of prostate cancer) [26,27] constitute the only in vivo pharmacokinetic study of a Pd(II)-compound carried out to date. However, this agent has been designed to exert local pharmacological effect only upon activation with a specific wavelength laser light, which produces reactive oxygen species in the target area. Therefore, its utility for the pharmacokinetic comparisons to Pd_2_Spm is limited. The present work aimed to provide insights into the in vivo pharmacokinetics and biodistribution of a dinuclear Pd(II)-spermine complex (Pd_2_Spm) as compared to the mononuclear Pt(II) drug cisplatin. Additionally, due to the promising anticancer activity of Pd_2_Spm towards triple-negative breast cancer cells (as previously reported by the authors [19,20]), the in vitro cellular uptake of Pd_2_Spm and cisplatin in triple-negative breast cancer cells was also assessed to address the differences in intracellular accumulation of these two compounds. The amount of total Pd or Pt in cancer cells and biologic samples (serum, serum ultrafiltrate, kidney, liver, brain, heart, lungs, ovaries, mammary glands and white adipose tissue) was determined by inductively coupled plasma-mass spectrometry (ICP-MS), which enables highly sensitive measurements of metal-based compounds (both the parent compound and its metabolites).

## 2. Results

### 2.1. Drug Pharmacokinetics in Serum and Serum Ultrafiltrate 

**Figure 2:**
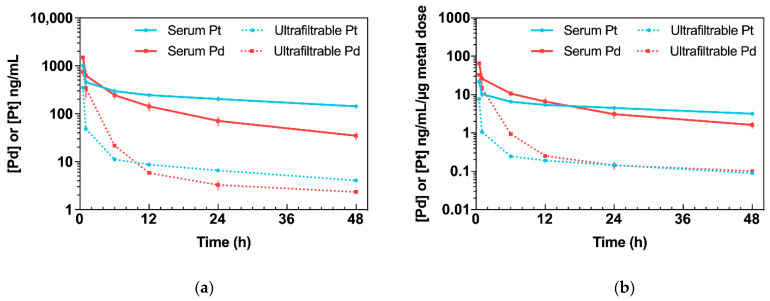
Kinetic profiles of Pd (from Pd_2_Spm, 3 mg/kg bw) and Pt (from cisplatin, 3.5 mg/kg bw) in serum and serum ultrafiltrate after single intraperitoneal bolus injection in Balb/c mice. Data are expressed as mean ± SEM (*n* = 5 animals per time point). (**a**) Concentration–time plot of mean Pd or Pt levels in serum and serum ultrafiltrate. (**b**) Concentration–time plot of mean Pd or Pt levels in serum and serum ultrafiltrate normalized to administered metal dose.

The concentrations of Pd and Pt in serum (total Pd/Pt) and serum ultrafiltrate (free Pd/Pt) after a single intraperitoneal bolus injection of Pd_2_Spm 3 mg/kg or cisplatin 3.5 mg/kg, in healthy female Balb/c mice, were measured by ICP-MS, the corresponding pharmacokinetic parameters obtained with non-compartmental analysis being summarized in Table 1. The serum concentration–time profiles of Pd (from Pd_2_Spm) and Pt (from cisplatin) are shown in Figure 2. The decrease in Pd and Pt concentrations in serum and serum ultrafiltrate showed a biphasic kinetic behavior with a rapid initial distribution phase followed by a prolonged first-order elimination phase in both Pd_2_Spm- and cisplatin-treated animals (see Figure 2). Since the maximum concentration (c_max_) for both Pd and Pt in plasma was reached immediately at the first sampling time-point, 0.5 h, the Pd/Pt concentration during the preceding period (<30 min post-injection) may be higher.

Pd_2_Spm administration (3 mg/kg bw) resulted in mean serum Pd concentrations within the range of 34.7 to 1499.4 ng/mL. The maximum concentration (C_max_), 1499.4 ± 38.5 ng/mL, declined rapidly within the first hour and decreased steadily during the terminal elimination phase. In serum, Pd was found to display an elimination half-life of 20.7 h and a clearance (CL/F) of 152.8 mL/h/kg. Its volume of distribution (Vz/F: 4555.0 mL/kg) in mice was greater than total body water, suggesting the widespread distribution of Pd in tissues. Free Pd reached C_max_ = 749.0 ± 120.6 ng/mL, with an elimination half-life of 35.5 h and CL/F = 858.8 mL/h/kg. At 48 h, the concentration of free Pd in serum ultrafiltrate was 2.3 ng/mL.

Administration of cisplatin (3.5 mg/kg) resulted in mean serum Pt concentrations within the range 142.4 to 1009.6 ng/mL. The C_max_ 1009.6 ± 64.2 ng/mL decreased steadily with a terminal half-life of 43.3 h and a CL/F of 115.4 mL/h/kg. The volume of distribution was 7202.8 mL/kg. Free Pt in serum peaked at a maximum concentration of C_max_ = 350.7 ± 16.8 ng/mL, with an elimination half-life of 31.5 h, a volume of distribution equal to 143,864.7 mL/kg and CL/F = 2989.9 mL/h/kg. At 48 h, the concentration of free Pt was found to be 4.1 ng/mL.

Even though the doses of Pd_2_Spm (3 mg/kg) and cisplatin (3.5 mg/kg) used in this study were somewhat similar, the actual dose of Pd administered to the animals was only half of the dose of Pt owing to the difference in the metal composition in each compound (dinuclear versus mononuclear in Pd_2_Spm and cisplatin, respectively; please see Section 4.8, for further clarification). Therefore, to obtain biologically meaningful comparisons of relative metal kinetics, the mean Pd and Pt concentrations were normalized to the administered metal dose (Figure 2b). Regarding the metal dose-normalized concentration–time profiles in serum ultrafiltrate, a higher concentration of free Pd than Pt was found during the 0.5–12 h period, reaching equivalent concentrations for both metals during the 24–48 h period. The dose normalization also revealed that animals treated with Pd_2_Spm reached a 3- and a 4.2-fold higher C_max_ in serum and serum ultrafiltrate, respectively, compared to cisplatin. Additionally, considering the dose-normalized areas under the curve (AUCs), Pd_2_Spm administration resulted in a 1.2- (*p* > 0.05) and a 4.3-fold (*p* < 0.001) higher AUC for Pd in serum and serum ultrafiltrate when compared to cisplatin, respectively. 

### 2.2. Drug Binding to Plasma Proteins

**Figure 3:**
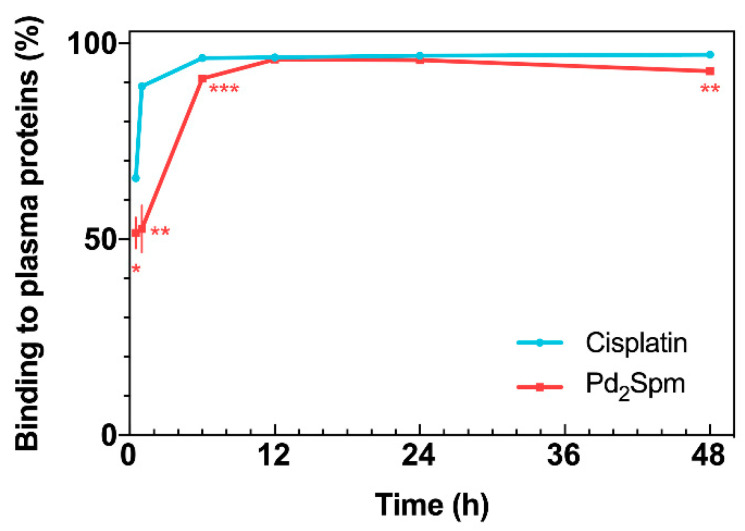
Drug binding to plasma proteins versus time, for cisplatin and Pd_2_Spm. Data are expressed as mean ± SEM (*n* = 5 animals per time point) and were compared with Student’s *t*-test. * *p* < 0.05, ** *p* < 0.01, *** *p* < 0.001.

The analysis of Pd and Pt concentrations in serum ultrafiltrate showed different profiles of plasma protein binding. At 0.5 and 1 h following Pd_2_Spm administration, only ca. 50% of Pd from Pd_2_Spm was bound to plasma proteins and 91.0% at 6 h, reaching the maximum protein plasma binding of 95.9% at 12 h post-administration (Figure 3). At 48 h, 92.8% of Pd_2_Spm was bound to plasma proteins. For cisplatin, in turn, a faster binding was determined—65.5% at 0.5 h and 89.5% at 1 h, reaching a maximum of ca. 97% at 6 h (i.e., virtually twice as fast relative to Pd_2_Spm). A comparative analysis revealed significantly lower Pd binding (from Pd_2_Spm) to plasma proteins than Pt (from cisplatin) during the 0.5–6 h period after drug administration. Additionally, while at 12 and 24 h, Pd and Pt showed similar binding to plasma proteins (ca. 96–97%), at 48 h, the fraction of Pd bound to plasma proteins presented a 3.1% decrease compared to the preceding period.

### 2.3. Drug Tissue Biodistribution

**Figure 4:**
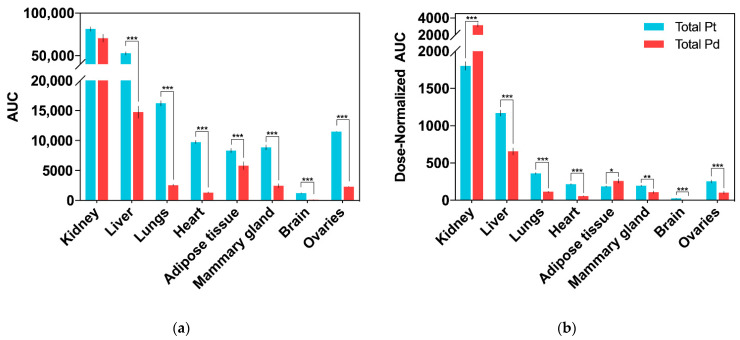
Biodistribution of total Pd (from Pd_2_Spm, 3 mg/kg bw) and Pt (from cisplatin, 3.5 mg/kg bw) in tissues after single intraperitoneal bolus injection in Balb/c mice. Data are expressed as mean ± SEM (*n* = 5 animals per time point) and were compared with Student’s *t*-test. * *p* < 0.05, ** *p* < 0.01, *** *p* < 0.001. (**a**) AUC_0-48h_ (area under the Pd/Pt concentration–time curve). Units: h × ng/g of tissue; (**b**) dose-normalized AUC_0–48 h_ (area under the Pd/Pt concentration–time curve). Units: h × ng/g per µg metal dose.

**Figure A1:**
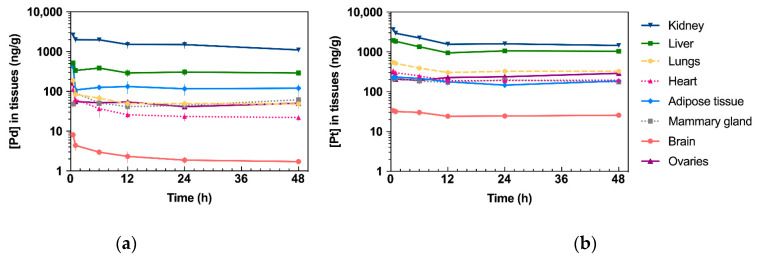
Kinetic profiles of Pd (from Pd2Spm, 3 mg/kg bw) and Pt (from cisplatin, 3.5 mg/kg bw) in tissues after single intraperitoneal bolus injection in Balb/c mice. Data are expressed as mean ± SEM (n = 5 animals per time point). Units: h.ng/g of tissue (**a**) Concentration–time plot of mean Pd levels in tissues. (**b**) Concen-tration–time plot of mean Pt levels in tissues.

**Figure A2:**
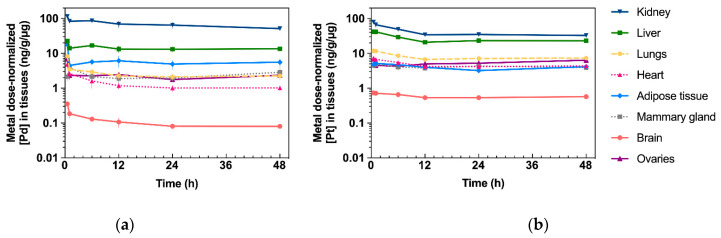
Metal dose-normalized kinetic profiles of Pd (from Pd_2_Spm, 3 mg/kg bw) and Pt (from cisplatin, 3.5 mg/kg bw) in tissues after single intraperitoneal bolus injection in Balb/c mice. Data are expressed as mean ± SEM (*n* = 5 animals per time point). Units: h.ng/g per µg metal dose (**a**) Concentration–time plot of mean Pd levels (normalized to administered metal dose) in tissues. (**b**) Concentration–time plot of mean Pt levels (normalized to administered metal dose) in tissues.

Accumulation of Pd (from Pd_2_Spm) and Pt (from cisplatin) in mice kidney, liver, lungs, heart, adipose tissue, mammary gland, brain and ovaries was determined by ICP-MS, and the corresponding AUC_0–48 h_ for each tissue is presented in Figure 4a. Except for the brain, Pd was widely distributed in the tested tissues, and the primary accumulation was found in the kidney, followed by the liver, lungs, ovaries, adipose tissue and mammary glands. Excluding the kidney, the animals treated with a single cisplatin dose showed a higher accumulation of Pt in all the organs than the Pd levels found in the organs of Pd_2_Spm-treated animals. In the kidney, similar Pd and Pt concentrations were found (when comparing cisplatin- and Pd_2_Spm-treated mice), suggesting a similar accumulation of metals in renal tissue when doses of 3 mg/kg of Pd_2_Spm and 3.5 mg/kg of cisplatin are administered. The corresponding kinetic profiles of Pd and Pt concentrations in tissues are also available in Appendix B—Figure A1. Since these metal concentrations at 12 h post-administration of either drug—Pd_2_Spm (Figure A1a) or cisplatin (Figure A1b) did not show a substantial decrease during the sampling period of 48 h, the pharmacokinetic parameters for each tissue could not be estimated. The normalization of AUCs to the administered metal dose evidenced 1.7- and 1.4-fold higher exposures to Pd versus Pt in kidney and adipose tissues, respectively, suggesting a higher relative accumulation of Pd_2_Spm. In turn, the dose-normalized AUCs for Pd in liver, lungs, heart, ovaries and mammary tissue were lower compared to Pt exposure; Pd was decreased by 43.9% in liver, 68.4% in lungs, 73% in heart, 44.1% in mammary tissue and 59.4% in ovaries as compared to Pt. The brain was the least exposed studied tissue, evidencing 82.7% lower accumulation of Pd than Pt, which indicates a reduced permeability of the blood–brain barrier to Pd_2_Spm than to cisplatin. The dose-normalized kinetic profiles for Pd (Figure A2a) and Pt (Figure A2b) in mice tissues are available in Appendix B.

### 2.4. In Vitro Cellular Drug Uptake

**Figure 5:**
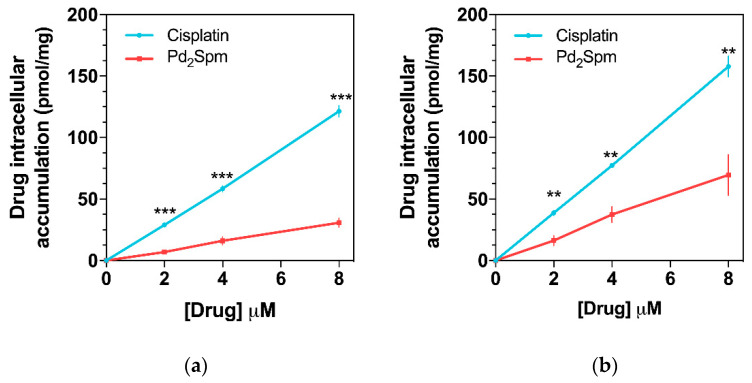
Cellular accumulation of Pd_2_Spm and cisplatin in (**a**) MDA-MB-231 and (**b**) HCC-1143 triple-negative breast cancer cells. Data are expressed as mean ± SEM (*n* = 4 independent experiments) and were compared with Student’s *t*-test. ** *p* < 0.01, *** *p* < 0.001.

Since Pd_2_Spm has already shown promising in vitro anticancer activity against several cisplatin-resistant cancers [19,28], the in vitro cellular uptake/accumulation of either Pd_2_Spm or cisplatin in triple-negative breast cancer cells—MDA-MB-231 (from pleural metastasis of breast carcinoma) and HCC-1143 (from invasive ductal breast carcinoma)—was also evaluated by ICP-MS. The results of Pd_2_Spm or cisplatin accumulation in cells treated with equimolar drug concentrations (between 2 and 8 µM) are depicted in Figure 5 and evidenced a lower intracellular Pd_2_Spm increase as compared to cisplatin for both MDA-MB-231 and HCC-1143 cells (Figure 5a,b, respectively). This cellular drug uptake was shown to increase linearly with drug concentration in the cell culture medium, suggesting passive transport as the primary mechanism of cellular uptake for the two compounds. Interestingly, a higher accumulation of Pd_2_Spm (and cisplatin) was observed in HCC-1143 cells relative to MDA-MB-231 cells.

## 3. Discussion

In the present study, the pharmacokinetics and biodistribution of Pd(II)-spermine chelate Pd_2_Spm were investigated after a single intraperitoneal bolus injection in Balb/c mice. Biphasic kinetics was observed upon the administration of Pd_2_Spm (3 mg/kg), with similar pharmacokinetics and tissue biodistribution patterns to those of the reference drug, cisplatin. Cisplatin´s pharmacokinetics (at a 3.5 mg/kg dose) was determined in parallel, with the results agreeing with previously published studies [29,30,31].

Even though the terminal half-life of Pd (from Pd_2_Spm) in serum was found to be 20.7 h, in contrast with 40.3 h for Pt (from cisplatin), the free Pd in serum ultrafiltrate had a higher terminal half-life than Pt (35.5 versus 31.5 h, respectively), which suggests a longer persistence of free Pd in blood circulation during the terminal elimination phase. The analysis of serum ultrafiltrate has particular relevance since this biological matrix contains free drug and its low molecular weight metabolite(s) (<30 kDa in this study prepared with a cut-off 30K NMWL filter), which are both responsible for exerting pharmacological effects [32]. Upon systemic administration, the drug occurs in plasma in different forms: (i) as a free, intact molecule administered in vivo; (ii) free hydrolyzed species and/or low molecular weight metabolites; and (iii) a plasma protein-bound drug (either via reversible or irreversible bonds with different affinities and kinetics). The drug binding to plasma proteins (namely, albumin, α1-acid glycoprotein and lipoproteins) is an unavoidable effect that influences the pharmacokinetic and pharmacodynamic properties of all systemically administered drugs. In general, a drug´s high binding to plasma proteins results in the drug´s lower tissue permeability, a small volume of distribution, decreased metabolism, slower clearance and prolonged half-life. In turn, considering the widely accepted free drug theory, the concentration of the free drug in plasma correlates more accurately with the drug´s pharmacological effects and toxicity than the total plasma concentration (free drug plus protein-bound drug), as only the free drug is able to reach its target in tissues and exert therapeutic effects. The free drug theory also stipulates that when a steady-state equilibrium is reached in the plasma, the free drug concentration in plasma reflects the pharmacologically relevant amount of the free drug at the target site (i.e., tissues) [33]. Regarding conventional Pt(II)-agents (cisplatin, carboplatin and oxaliplatin), previous in vivo studies in rodents have shown different interactions of these drugs with plasma proteins; at 3 h post-administration, >85% of cisplatin and oxaliplatin were bound to albumin mostly via irreversible covalent bonds, while only 20% to 50% of carboplatin was reversibly bound to plasma proteins [34,35,36]. Indeed, Pt-based chemotherapeutics undergo both aquation reactions and biologic transformation in blood, which allow the binding of a drug’s metal center to cysteine and methionine residues of amino acids that constitute plasma proteins (mostly via covalent bonds that inactivate platinum agents) [37]. This process is highly time-dependent and differs between different platinum drugs, depending on the stability of its ligands and leaving groups [38]. In the present work, the analysis of serum ultrafiltrate samples showed that at 1 h post-administration, only ca. 50% of Pd_2_Spm was bound to plasma proteins compared to ca. 90% for cisplatin, revealing different affinities of Pd_2_Spm and cisplatin towards plasma proteins. Since most cisplatin is bound to plasma proteins via irreversible bonds, only a small fraction of administered cisplatin is available to cross cell membranes and exert pharmacological effects. Pd_2_Spm, on the other hand, showed a lower affinity to coordinate to plasma proteins, which indicates that following administration, the majority of Pd_2_Spm is initially present in the plasma as a free unbound drug, which may more easily reach its targets in the tissues and exert pharmacological effects. These results are further supported with 4.3-fold (*p* < 0.001) higher AUC and longer elimination half-life (35.5 h) of free Pd in serum ultrafiltrate, which suggests a higher availability and persistence of free Pd than free Pt in blood circulation.

Concerning the drug´s in vivo biodistribution, both Pd_2_Spm and cisplatin showed a similar pattern in the five major organs analyzed (brain, lungs, heart, liver and kidney). The highest exposure to Pd and Pt was observed in the kidney, followed by the liver, lungs, heart and brain, with the latter being the least exposed organ. However, despite the similar affinity for accumulation in specific organs, the comparative analysis of relative metal amount in tissues (measured as metal dose-normalized AUC) revealed a significantly lower exposure to Pd in the liver, lungs, heart and brain as compared to Pt. Therefore, owing to its limited accumulation, Pd_2_Spm is expected to be well tolerated, without noteworthy deleterious effects in these organs. Additionally, our data suggest that the low accumulation of Pd in the brain may be explained by the very low permeability of the blood–brain barrier to Pd_2_Spm; thus, this agent is highly unlikely to affect the central nervous system. In turn, a 74.2% higher accumulation of Pd (from Pd_2_Spm) relative to Pt (from cisplatin) was determined in the kidney. This very large disparity in the accumulation of Pd and Pt in the kidney compared to the other organs was an expected outcome, which agrees with previous reports for several Pt-based compounds [39,40,41]. The dose-dependent nephrotoxicity of Pt-based drugs results from the binding of the drug´s Pt center to thiol-containing renal enzymes (e.g., metallothioneins) [24] but, as shown for oxaliplatin and carboplatin, which displayed a higher and lower renal accumulation than cisplatin, respectively [42], the total content of Pt accumulated in the kidney is an unreliable biomarker of nephrotoxicity, and attention should be paid to specific interactions between the drug/its metabolites and renal biomolecules likely to be responsible for functional deviation and associated toxicity [36,42,43]. Several authors have anticipated lower nephrotoxicity elicited by Pd-based compounds owing to their decreased reactivity towards sulfhydryl groups of proteins in the kidney; however, it still remains to be fully understood under in vivo conditions [25,44]. Accordingly, since we observed the highest exposure to Pd_2_Spm (and cisplatin) in the kidney and liver, the metabolic impact of Pd_2_Spm and cisplatin on these organs had already been the subject of our parallel work aiming to determine possible deleterious alterations [45,46]. Using an identical experimental design, the metabolomics results evidenced that Pd_2_Spm treatment triggered rapid and short-term changes with almost complete recovery to control levels in the liver (both polar and lipid metabolites) and kidney (polar metabolites), while cisplatin induced distinctive and more persistent deviations [45]. In general, these metabolic results provided encouraging evidence that Pd_2_Spm treatment may induce lower negative effects compared to the treatment with cisplatin [46], despite considerable Pd accumulation in the kidney and liver.

Finally, the comparative study of the cellular uptake of either Pd_2_Spm or cisplatin in MDA-MB-231 and HCC-1143 breast cancer cells showed that the in vitro accumulation of these drugs within the 2 to 8 µM range increases linearly with concentration and appears to follow Fick’s law for passive diffusion. Interestingly, a higher accumulation of Pd_2_Spm (as well as of cisplatin) was observed in HCC-1143 cells relative to MDA-MB-231 cells, which is consistent with the putative occurrence of different uptake mechanisms for these two types of cell lines, which may also differ in the cell membrane composition, as previously reported for several breast cancer cell lines [47]. While a lower intracellular uptake of Pd_2_Spm was observed compared to cisplatin, Pd_2_Spm has been reported to cause more extensive phosphorylation of H2AX histone (γH2AX) [28], which is an indirect measure of DNA breaks induced by metalation of the nucleic acid. This result is in agreement with previously reported data on the mode of action of this polynuclear agent—Pd_2_Spm is able to bind to DNA via unconventional, long-range crosslinks, which are more deleterious than conventional interactions (e.g., cisplatin-prompted), particularly towards fast-growing malignant cells [17,22,23]. A similar discrepancy between desired pharmacologic effects and cellular uptake has been recently reported for conventional Pt-based agents—cisplatin, carboplatin and oxaliplatin [5]. This study by Schoch and co-workers (2020) showed that carboplatin shares a mechanism of action with cisplatin, while oxaliplatin acts differently: the former led to identical DNA lesions (and a comparable gene response), and the latter exhibited similar cytotoxicity to cisplatin, despite its lower intracellular accumulation [5]. Therefore, since Pd_2_Spm showed linear accumulation in breast cancer cells coupled to favorable pharmacokinetic and biodistribution properties, this study provides an encouraging basis for further in vivo studies focused on the anticancer properties of Pd_2_Spm. While the current study provides valuable findings regarding the pharmacokinetics and biodistribution of Pd (from Pd_2_Spm) in serum, serum ultrafiltrate and tissues, the excretion/metabolism of Pd_2_Spm was not addressed in this work and will be investigated in future research.

## 4. Materials and Methods

### 4.1. Reagents and Chemicals

Polypropylene 10 mL tubes with high-density polyethylene screw caps (Ref# 62.9924.284) used for sample digestions were from Sarstedt (Nümbrecht, Germany). Nitric acid (HNO_3_, TraceSELECT™, ≥69.0%) and hydrochloric acid (HCl, TraceSELECT™, ≥30%) were purchased from Honeywell Fluka™ (Düsseldorf, Germany). For ICP-MS determinations, the multi-element standard solution TraceCERT^®^ (100 mg/L), ICH Q3D oral, Standard 2 solution from Merck KGaA (Darmstadt, Germany) and the internal standard solution ICP-MS-200.8-IS-1 (100 mg/L of Sc, Y, In, Tb, Bi) from AccuStandard^®^ (New Haven, CT, USA) were used. Ultrapure water (18.2 MΩ.cm at 25 °C) was obtained with an Arium^®^ pro water purification system (Sartorius, Goettingen, Germany). Animals were euthanized with Euthasol^®^ solution (400 mg/mL pentobarbital sodium) acquired from Le Vet (Oudewater, The Netherlands). Cisplatin (cis-dichlorodiammine platinum(II), 99.9%), potassium tetrachloropalladate (II) (K_2_PdCl_4_, 98%), spermine (N,N’-bis(3-aminopropyl)-1,4-diaminobutane, 99%), DMEM-HG cell culture medium and RPMI-1640 cell culture medium were purchased from Sigma-Aldrich (Sintra, Portugal). Fetal bovine serum (FBS) was from Gibco (Thermo Fisher Scientific, Inc., Waltham, MA, USA). All the other reagents were of analytical grade.

### 4.2. Cell Culture

The in vitro studies were performed on human triple-negative (lack of estrogen receptors, progesterone receptors and HER2 overexpression) breast cancer cell lines MDA-MB-231 and HCC-1143, which were purchased from the Leibniz Institute DSMZ-German Collection of Microorganisms and Cell Cultures GmbH, Germany. MDA-MB-231 were cultured in DMEM-HG cell culture medium supplemented with 10% (*v*/*v*) FBS, and HCC-1143 cells were cultured in RPMI-1640 cell culture medium supplemented with 20% (*v*/*v*) FBS. Cells were maintained in a sterile environment at 37 °C with a 5% CO_2_ humidified atmosphere and were routinely tested for mycoplasma contamination.

### 4.3. Ethical Considerations

The European Directive 2010/63/EU on the protection of laboratory animals used for scientific purposes (European Parliament, Council of the European Union, 2010) and the Portuguese law on animal welfare (Decreto-Lei 113/2013) were followed for designing and conducting all research procedures. The study protocol was approved by the Ethics Committee of the Faculty of Pharmacy of the University of Porto, Porto, Portugal (Permit Number: 25-10-2015), and by the Ethics Committee and the Organ Responsible for the Welfare of Animals of ICBAS-UP, Porto, Portugal (Permit number 134/2015). All sections of this study follow the ARRIVE Guidelines for reporting animal research [48].

### 4.4. Animals

Six-week old, Specific-Pathogen-Free (SPF), female BALB/cByJ mice (60 animals in total) were purchased from Charles River Laboratories (L’Arbresle, France) and acclimatized at ICBAS-UP Rodent Animal House Facility (Porto, Portugal) for one week. Animals were randomly distributed into groups of five per individually ventilated cage and were housed under controlled SPF environmental conditions (temperature 22.5 ± 1.5 °C; relative humidity 50 ± 10%; 12 h light/dark cycle) with ad libitum access to water and standard pellet food (4RF21, Mucedola, Italy). Environmental enrichment included corncob bedding, paper roll tube and one large sheet of tissue paper for nesting. Animals were monitored daily for health status, and no adverse events were observed during the study.

### 4.5. Pd_2_Spm Synthesis and Formulation

Pd_2_Spm complex was synthesized according to published procedures [49] optimized by the authors [50]: 2 mmol of K_2_PdCl_4_ was dissolved in a minimal amount of water, and 1 mmol of spermine aqueous solution was added dropwise under continuous stirring. After 24 h, the resulting orange powder was filtered, washed with acetone, and yellow-orange needle-shaped crystals were recrystallized from water. The composition and purity of synthesized compound were fully characterized by elemental analysis and vibrational spectroscopy (FTIR, Raman and inelastic neutron scattering), which were compared with the previously calculated vibrational profiles (by ab initio methods) [50,51]. Yield: 68%. Elemental analysis (Pd_2_(C_10_N_4_H_26_)Cl_4_): found—C: 21.2%; H: 4.7%; N: 9.6%, Cl: 25.9%; calculated—C: 21.5%; H: 4.6%; N: 9.9%, Cl: 25.6%. Pd_2_Spm 0.3 mg/mL solution for in vivo administration was freshly prepared by dissolving an appropriate quantity of drug in Phosphate-Buffered Saline (PBS) (H_2_PO_4_ 1.5 mM, Na_2_HPO_4_ 4.3 mM, KCl 2.7 mM, NaCl 150 mM, pH 7.4) containing 1% of DMSO and sterile-filtered. The solution of cisplatin 0.35 mg/mL was prepared in PBS and sterile-filtered.

### 4.6. Pharmacokinetic Study Design and Sample Collection

Animals were randomly allocated into two study groups (30 animals/group): Pd_2_Spm and cisplatin. No control group was used since Pt and Pd metals were inexistent in drug-naïve rodents. On the day of the experiments, animals were weighed (mean ± SD: 20.4 ± 1.7 g), and a single dose of Pd_2_Spm 3 mg/kg BW (corresponds to 1.15 mg/kg BW of Pd) or cisplatin 3.5 mg/kg BW (corresponds to 2.28 mg/kg BW of Pt) was administered via intraperitoneal bolus injection (200 µL/injection) in Pd_2_Spm and cisplatin groups, respectively. After drug administration, five animals from each group were euthanized at selected time-points (0.5, 1, 6, 12, 24 and 48 h) with pentobarbital intraperitoneal injection (120 mg/kg) followed by cardiac puncture for blood collection. Blood serum was separated after leaving the blood to coagulate for 30 min at room temperature and centrifugation (at 500× *g*, and 4 °C for 15 min). The serum was separated, and a small aliquot was filtered using Amicon Ultra-0.5 mL Centrifugal Filters (14,000× *g*, 20 °C, 30 min) with 30K NMWL (Merck KGaA, Darmstadt, Germany) to obtain serum ultrafiltrate for the study of drug binding to plasma proteins. Both blood serum and serum ultrafiltrate were aliquoted (50 μL) and stored at −80 °C until ICP-MS analysis. Right brain hemisphere, liver, lungs, heart, right kidney, ovaries, abdominal white adipose tissue and abdominal mammary glands were excised at the same time, washed in cold PBS, blotted dry on filter paper and weighed. Solid tissues were freeze-dried (0.016 mBar, −80.8 °C, 48 h; Telstar^®^, model Cryodos Barcelona, Spain) and stored hermetically sealed at −80 °C until ICP-MS analysis. The complete characterization of the study groups with the respective body and wet tissue weights are available in Appendix A—Figure A1.

### 4.7. Sample Preparation and ICP-MS Analysis

Quantification of Pd and Pt in the biologic samples collected from in vivo pharmacokinetic studies was achieved using a validated method previously developed by our group [52]. Briefly, 50 μL of blood serum, 50 μL of serum ultrafiltrate and weighed entire freeze-dried organs/tissues (up to 50 mg) were placed into 10 mL polypropylene conical tubes. In the case of liver, for which the weight surpassed 50 mg, the tissue was first pulverized with a Precellys^®^ Evolution tissue homogenizer (Bertin-Instruments, Montigny-le-Bretonneux, France) using 2.8 mm stainless steel beads (two cycles of 10 s at 6000 rpm speed) and accurately weighed (50 mg) in tubes. Samples were digested directly in tubes with 0.9 mL of HNO_3_ (≥69.0% *w*/*w*) plus 0.3 mL of HCl (37% *w*/*w*) in a water bath at 90 °C for 1 h. Sample digests were cooled to room temperature, 0.5 mL of a 100 µg/L internal standards (IS) solution was added and the final volume was adjusted to 5 mL with ultrapure water (final concentration of IS = 10 µg/L). Prior to ICP-MS analysis, samples were further diluted 5-fold with a diluent solution (2% HNO_3_ + 3% HCl + 10 µg/L IS). An iCAP^TM^ Q (Thermo Fisher Scientific, Bremen, Germany) ICP-MS was used for the determination of Pd and Pt in the obtained solutions. Calibration standards ranging from 0.025 to 100 µg/L were prepared from the 100 mg/L multi-element commercial TraceCERT^®^, ICH Q3D oral, Standard 2 solution. The IS solution was prepared at 100 µg/L by appropriate dilution of the ICP-MS-200.8-IS-1 solution. The elemental isotopes (*m/z* ratios) ^108^Pd and ^195^Pt were used for analytical determination. ^89^Y, ^115^In, ^159^Tb and ^209^Bi were used as internal standards.

### 4.8. Pharmacokinetic Analysis

Pharmacokinetic parameters for Pd_2_Spm and cisplatin were estimated using Phoenix WinNonlin v8.3.1.5014 (Certara USA Inc., Princeton, NJ, USA). The data were expressed as nanograms of Pd (from Pd_2_Spm) or Pt (from cisplatin) measured by ICP-MS per gram of wet tissue weight or per mL of serum/serum ultrafiltrate. Considering the molecular weights of each compound and the molar ratios of respective atoms, the molar ratio of Pd atom in Pd_2_Spm molecule is 2:1 with mass Pd (%) = 38.21%, [MW(Pd_2_Spm) = 557 g/mol]; and the molar ratio of Pt atom in cisplatin molecule is 1:1 with mass Pt (%) = 65.02%, [MW(cisplatin) = 300.01 g/mol]. Accordingly, the administered dose of Pd (from Pd_2_Spm 3 mg/kg) was 1.15 mg/kg, and the administered dose of Pt (from cisplatin 3.5 mg/kg) was 2.28 mg/kg. The mean concentrations of Pd or Pt in serum and serum ultrafiltrate were plotted logarithmically versus time. Non-compartmental analysis was performed on sparse data with 1/Y^2^ weighting, which was selected based on the highest correlation coefficients and agreement between observed versus predicted pharmacokinetic parameters. The highest drug concentration observed following administration (C_max_) and the time at which it was observed (t_max_) were established from the temporal evolution of respective Pd/Pt concentrations in serum and serum ultrafiltrate. The area under the curve (AUC) from time zero to the last time with quantified concentration (AUC_0-48h_) was calculated by a linear log trapezoidal (linear up log down) method. The AUC from time zero to infinity (AUC_0−∞_) was extrapolated as AUC_0−48 h_ + C_48h_/λz, where C_48h_ is the last measured concentration, and λz is the elimination constant rate calculated using linear regression of the terminal portion of the log–linear serum concentration–time curve. The drug half-life (t_1/2_) was calculated as ln2/λz. The rate of total clearance (CL/F) of the compound was calculated as the administered metal dose divided by the AUC_0−∞_. The distribution volume based on the terminal phase (Vz/F) was calculated as dose/[λz.AUC_0−∞_].

### 4.9. Intracellular Accumulation of Pd_2_Spm or Cisplatin in Breast Cancer Cells

The intracellular Pt and Pd accumulation in MDA-MB-231 and HCC-1143 cells was analyzed using ICP-MS. First, 3 × 10^4^/cm^2^ cells were seeded in T75 culture flasks and allowed to attach for 24 h. Cells were subsequently treated for 24 h with 2, 4 or 8 μM of either Pd_2_Spm or cisplatin in four independent experiments (*n* = 4). The cells were trypsinized, washed twice with ice-cold 0.9% saline solution, and 10^6^ cells were pelleted and freeze-dried. The lyophilized cell pellets were digested and analyzed using the same analytical procedure described for the pharmacokinetic study. The quantity of accumulated Pd and Pt in cells was converted in the correspondent concentration of Pd_2_Spm and Cisplatin in pmoles per 1 mg of protein. The protein concentration used for the data normalization was determined from identical samples that were prepared in parallel and quantified with the Bradford protein assay.

### 4.10. Statistical Analysis

Data are expressed as the mean ± standard error of the mean (SEM) and compared with a two-tailed Student’s t-test, using GraphPad Prism 7 Software (San Diego, CA, USA). A *p*-value < 0.05 was considered statistically significant.

## 5. Conclusions

Altogether, this work reported, for the first time, an in vivo pharmacokinetic study of a dinuclear Pd(II)-polyamine anticancer agent (Pd_2_Spm) after intraperitoneal bolus injection in healthy Balb/c mice. Using ICP-MS, the tissue biodistribution and several pharmacokinetic parameters were obtained and compared with those measured for cisplatin, taken as a reference drug. Furthermore, the in vitro cellular uptake/accumulation of Pd_2_Spm and cisplatin in triple-negative breast cancer MDA-MB-231 and HCC-1143 cells was also carried out to assess the differences in the transport mechanism and cellular accumulation of these two compounds.

Pd_2_Spm showed appropriate pharmacokinetics regarding its potential therapeutic application. In addition, it displayed a slower plasma protein binding and a higher terminal half-life of free Pd in serum as compared to cisplatin. Since the overall accumulation of Pd in tissues was lower than Pt (in Pd_2_Spm- and cisplatin-treated mice, respectively), it is expected that Pd(II)-agent might present decreased systemic adverse effects. Additionally, the in vitro assays evidenced an increase in the drug’s intracellular concentration with the applied dose, which implies a passive diffusion as a primary mechanism of cellular entry. In short, this study provides valuable insights into i) the in vivo pharmacokinetic profile and biodistribution of Pd_2_Spm and ii) the in vitro intracellular uptake/accumulation in triple-negative breast cancer cells, allowing further research and preclinical development of this promising compound. In addition, the results thus gathered constitute a reliable scientific basis for future pharmacokinetics studies of other Pd-based potential anticancer agents.

## Figures and Tables

**Table 1:** Pharmacokinetic parameters of total Pd and Pt in serum and serum ultrafiltrate following intraperitoneal bolus injection of Pd_2_Spm (3 mg/kg bw) and cisplatin (3.5 mg/kg bw) in Balb/c mice.

Pharmacokinetic Parameter (Unit)	Pd_2_Spm		Cisplatin	
	Total Pd in Serum	Free Pd in Ultrafiltrate	Total Pt in Serum	Free Pt in Ultrafiltrate
Half-life (h)	20.7	35.5	43.3	31.5
λ_z_ (1/h)	0.034	0.019	0.016	0.022
T_max_ (h)	0.5	0.5	0.5	0.5
C_max_ (ng/mL)	1499.4 ± 38.5	749.0 ± 120.6	1009.6 ± 64.2	350.7 ± 16.8
C_max_/Dose (ng/mL/µg)	65.9 ± 4.2	32.7 ± 5.3	21.9 ± 1.9	7.7 ± 0.4
AUC_0–48 h_ (h × ng/mL)	6490.2	1219.7	10867.5	567.2
AUC_0–48 h/_Dose (h × ng/mL/µg)	285.0	53.2	240.5	12.5
AUC_0–∞_ (h × ng/mL)	7523.9	1339.1	19,758.5	751.5
Vz/F (mL/kg)	4555.0	43,992.0	7202.8	143,864.7
CL/F (mL/h/kg)	152.8	858.8	115.4	2989.9

λz = elimination rate constant; T_max_ = time to reach C_max_; C_max_ = mean maximum concentration ± SEM; AUC_0−48 h_ = area under curve from time zero to 48 h; AUC_0−∞_ = area under the curve from time zero to infinity; Vz/F = volume of distribution at terminal phase; CL/F = body clearance.

## Data Availability

The data presented in this study are available on request from the corresponding author.

## Appendix A

**Table A1:** The characterization of the study groups with the weights of animals and respective wet tissue weights.

Drug	Time (h)	Body Weight (g)	Kidney (mg)	Liver (mg)	Lungs (mg)	Heart (mg)	Brain (mg)	Adipose Tissue (mg)	Mammary Gland (mg)
Ciplatin	0.5	20.6 ± 2.3	157.7 ± 19.4	962.3 ± 120.8	192.5 ± 31.5	110.9 ± 10.6	182.8 ± 28.0	101.8 ± 44.0	110.8 ± 31.1
	1	20.0 ± 1.6	151.8 ± 23.4	765.8 ± 378.5	159.0 ± 32.9	99.5 ± 7.6	178.0 ± 29.4	95.0 ± 46.7	81.5 ± 37.1
	6	19.6 ± 0.9	140.4 ± 10.1	824.5 ± 144.3	197.1 ± 7.2	104.8 ± 7.5	188.9 ± 22.3	86.6 ± 30.9	100.3 ± 45.3
	12	20.0 ± 2.1	156.3 ± 20.2	878.3 ± 224.3	184.3 ± 23.5	114.4 ± 19.0	166.9 ± 34.9	93.8 ± 32.1	105.3 ± 44.4
	24	19.8 ± 1.5	153.6 ± 14.9	824.5 ± 155.8	173.0 ± 21.1	109.6 ± 5.6	183.3 ± 22.8	87.9 ± 28.5	95.0 ± 46.4
	48	19.8 ± 2.0	149.3 ± 20.2	939.7 ± 137.0	166.1 ± 17.2	107.6 ± 8.9	195.7 ± 24.6	79.3 ± 16.9	116.8 ± 21.2
Pd_2_Spm	0.5	21.2 ± 1.3	160.9 ± 11.2	897.9 ± 199.2	188.8 ± 16.8	113.4 ± 8.1	200.7 ± 31.7	82.6 ± 12.9	119.0 ± 12.4
	1	22.2 ± 1.1	161.2 ± 7.2	1034.4 ± 89.6	187.9 ± 27.8	117.0 ± 7.0	191.5 ± 20.7	87.7 ± 12.3	119.0 ± 18,6
	6	21.0 ± 1.2	153.3 ± 12.6	1056.8 ± 264.3	188.3 ± 18.9	112.7 ± 12.0	212.8 ± 23.4	86.8 ± 14.2	115.0 ± 23.3
	12	19.6 ± 2.2	148.9 ± 14.4	1010.1 ± 196.4	188.4 ± 11.9	105.3 ± 7.9	179.8 ± 18.7	89.3 ± 20.8	88.1 ± 21.7
	24	20.6 ± 1.1	168.4 ± 14.8	868.8 ± 76.0	190.6 ± 19.8	115.9 ± 0.4	190.1 ± 11.4	63.1 ± 17.3	83.0 ± 27.9
	48	20.6 ± 0.9	154.4 ± 6.3	983.9 ± 279.7	197.1 ± 21.0	105.5 ± 6.1	203.1 ± 24.6	68.6 ± 8.0	87.7 ± 26.3

Data are means of five animals per time point ± standard deviation (*n* = 5/time point).
